# Sequential Unfolding of Beta Helical Protein by Single-Molecule Atomic Force Microscopy

**DOI:** 10.1371/journal.pone.0073572

**Published:** 2013-08-29

**Authors:** David Alsteens, Nicolas Martinez, Marc Jamin, Françoise Jacob-Dubuisson

**Affiliations:** 1 Université catholique de Louvain, Institute of Condensed Matter and Nanosciences, Louvain-la-Neuve, Belgium; 2 Unit for Virus Host Cell Interactions, UMI3265 UJF-EMBL-CNRS, Grenoble, Franc; 3 Institut Laue Langevin, Grenoble, France; 4 Centre for Infection and Immunity of Lille, INSERM 1019, CNRS 8204, Université Lille-Nord de France, Lille, France; Swiss Federal Institute of Technology Zurich, Switzerland

## Abstract

The parallel βhelix is a common fold among extracellular proteins, however its mechanical properties remain unexplored. In Gram-negative bacteria, extracellular proteins of diverse functions of the large ‘TpsA’ family all fold into long βhelices. Here, single-molecule atomic force microscopy and steered molecular dynamics simulations were combined to investigate the mechanical properties of a prototypic TpsA protein, FHA, the major adhesin of *Bordetella pertussis*. Strong extension forces were required to fully unfold this highly repetitive protein, and unfolding occurred along a stepwise, hierarchical process. Our analyses showed that the extremities of the βhelix unfold early, while central regions of the helix are more resistant to mechanical unfolding. In particular, a mechanically resistant subdomain conserved among TpsA proteins and critical for secretion was identified. This nucleus harbors structural elements packed against the βhelix that might contribute to stabilizing the N-terminal region of FHA. Hierarchical unfolding of the βhelix in response to a mechanical stress may maintain β-helical portions that can serve as templates for regaining the native structure after stress. The mechanical properties uncovered here might apply to many proteins with β-helical or related folds, both in prokaryotes and in eukaryotes, and play key roles in their structural integrity and functions.

## Introduction

The specific functions of extracellular proteins impose constraints on their structural properties. Extracellular proteins have also evolved to withstand various forms of stress in order to maintain function in non-controllable conditions. In addition, those proteins adopt folds that are compatible with their transport across membranes. Those constraints probably account for the large proportion of repetitive extracellular proteins. βhelices are such repetitive proteins, composed of coils formed of three short, amphipathic βstrands connected by variable-length turn regions [1]–[3]. The strands of successive coils stack on top of one another, forming parallel βsheets with conserved hydrogen-bonding patterns [2]. The interior of βhelices is mostly hydrophobic and shielded from the milieu by capping structures at its extremities [4]. βhelices display extensive surface area well suited for binding, and their scaffold is also used for the presentation of specific functional determinants by the insertion of loops or domains. The βhelix and related solenoid folds – e.g. beta rolls, leucine-rich repeat proteins and spiral folds - have been adopted throughout the phylogenetic tree, from microbial and phage proteins to insulin-like growth factor receptors in higher eukaryotes and antifreeze proteins in insects [1], [2].

The β-helical fold is overrepresented among extracellular microbial proteins that mediate various interactions with the external environment. In particular, βhelices are extremely common among bacterial and fungal adhesins, phage proteins and extracellular polysaccharide-degrading enzymes [2], [5], [6]. Upon binding to target cells in the hosts or to specific substrates in the milieu, those proteins may be exposed to fluid flows or other types of mechanical stress. However, nothing is known on the mechanical properties of β-helical proteins. In contrast, for repetitive proteins that are composed of a string of small structural domains in tandem, such as titin and spectrin, the repetitive structure has been shown to form the basis for elasticity and mechanical resistance [7], [8]. β helices represent a very different structural paradigm, as they are essentially composed of a single, in some cases very long, domain.

**Figure 1:**
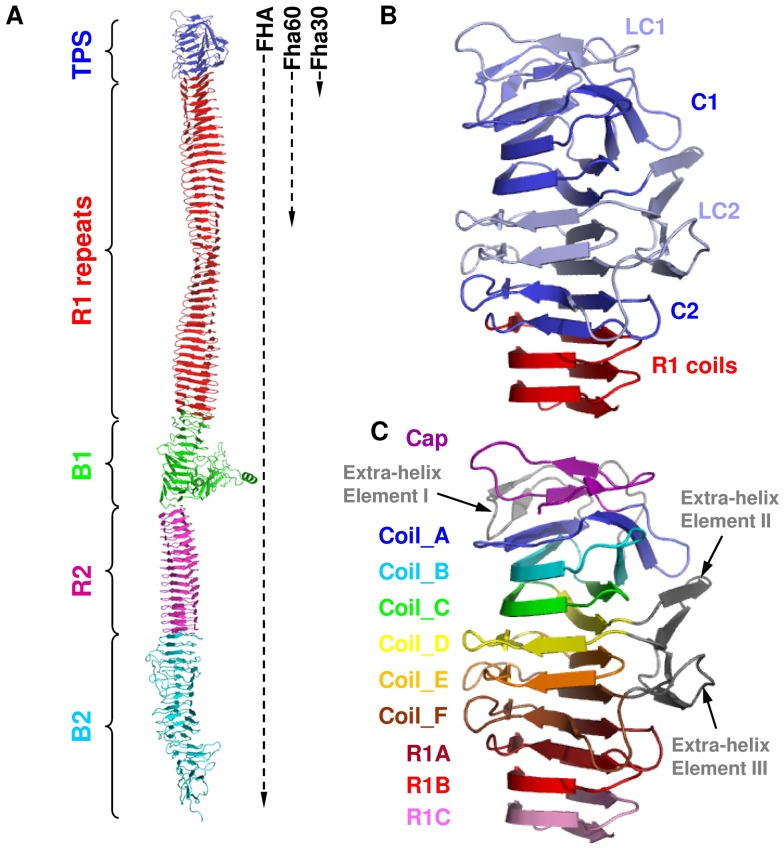
Schematic representation of FHA and model proteins used in this work. (A) Model of FHA. The dotted vertical lines at the right side of the protein model indicate the approximate lengths of Fha60 and Fha30, which both have the same N-terminus as full-length FHA. The TPS domain is shown in blue, the R1 region is shown in red, and the B1, R2 and B2 domains are shown in green, magenta and cyan, respectively. The X-ray structure of Fha30 is known, while the R1 and R2 regions were built by using the models reported in [18], and the B1 and B2 regions were built by molecular modeling using the I-TASSER web server [19]. (B) Conserved regions of Fha30. On the basis of multiple sequence alignments, four regions of different conservation rates were identified in the TPS domain. The most conserved subdomains C1 and C2 are shown in dark blue, and the less conserved subdomains LC1 and LC2 are shown in light blue. The first R1 coils are in red. (C) Structural organization of Fha30. The N-terminal cap and the six successive coils (Coil_A to Coil_F) of the TPS domain are shown in magenta, blue, cyan, green, yellow, orange and brown, respectively. The three R1 coils present in the crystal structure of Fha30 are shown in dark red, red and pink, and the three extra-helical elements are shown in grey. Elements II and III assemble together to form a four-stranded βsheet that packs against the β-helical core formed by coils_A to _F.

The first βhelix to have its structure determined was pectate lyase C (PelC) [3], a relatively small enzyme that contains seven complete βcoils. Since then, several other β?helical proteins have been characterized (e.g. [9]–[14]), which belong notably to two families of secreted bacterial proteins called TpsA proteins and autotransporters [6], [15]. Both families include proteins of several thousand residues predicted to form elongated βhelices. Filamentous haemagglutinin (FHA), the major adhesin of the whooping cough agent *Bordetella pertussis* [16], is a model TpsA protein. This 230-kDa protein appears by electron microscopy as a rigid, 40 nm-long rod [17]. It is mostly composed of approximately 80 imperfect sequence repeats of 19 residues or more in tandem that form the coils of an elongated βhelix, followed by a predicted globular domain at the C terminus [18] (Fig. 1A). FHA is secreted by the Two-Partner secretion (TPS) pathway [15] and is translocated across the outer membrane by its specific TpsB transporter, called FhaC [20]. Following translocation some FHA is found associated with the cell surface, consistent with its adhesive properties, although some of it is also found into the milieu, presumably exerting immunomodulatory effects [16]. FHA harbours a 250-residue-long N-terminal ‘TPS domain’, which is essential for secretion and conserved among TpsA proteins [12], [13], [21]. The structure of Fha30, a 30-kDa N-terminal fragment that represents the smallest secretion-competent FHA derivative has been determined [12] (Fig. 1B). Fha30 comprises the TPS domain followed by the first three regular ‘R1’ repeats of the central region of FHA, named R1A to R1C (Fig. 1C). The TPS domain forms a right-handed βhelix comprising six irregular coils called A to F that is capped by three β strands at the N terminus. The TPS domain also comprises three extra-helical elements, *i.e.* a β hairpin between coils_A and _B (extra-helix element I), another βhairpin within coil_D (extra-helix element II) and a βhairpin followed by a loop in coil_F (extra-helix element III). The βhairpins of the extra-helical elements II and III form a βsheet that packs against the βhelix (Fig. 1C). Based on sequence alignments of a number of TpsA proteins, the TPS domain has been subdivided into successive less conserved (LC) and conserved (C) regions called LC1, C1, LC2 and C2, respectively (Fig. 1B) [12]–[14], [21]. In this work, we used a combination of single-molecule AFM [22]–[24] and steered molecular dynamics (SMD) simulations to characterize the molecular elasticity of model FHA proteins. We show that FHA unfolds sequentially when subjected to force and that the TPS domain contains a stable substructure.

## Results

### Mechanical unfolding of TPS domain by single-molecule atomic force microscopy

FHA does not fold into its native conformation inside the cell, but only in the extracellular milieu after secretion [21]. Thus, attempts to produce intracellular FHA derivatives in a soluble form have been unsuccessful [21]. Furthermore, because the TPS domain is essential for secretion, only N-terminal FHA derivatives with the TPS domain can be prepared in a native form. Fha30 is the smallest secretion-competent FHA fragment [21]. Unlike the full-length protein, Fha30 and other short FHA truncates are essentially released from the cell surface. The FHA derivatives used in this work were thus secreted by the bacteria and purified from culture supernatants. In addition, the fusion of bulky domains to FHA derivatives prevents secretion [25], and thus only short tags were added to the extremities of the proteins under study.

**Figure 2:**
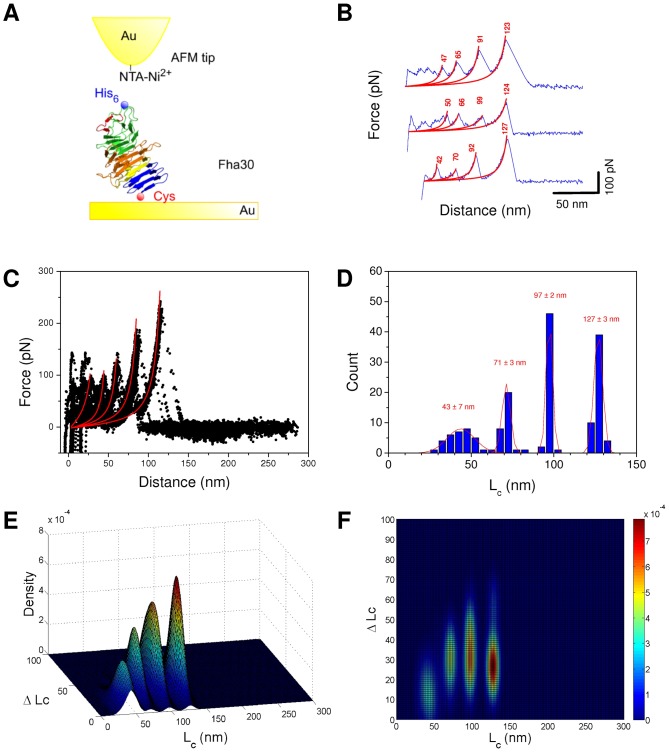
Unfolding of Fha30 by single-molecule AFM. (A) Experimental set-up. (B) Representative force-distance curves obtained by stretching a single polypeptide, showing periodic features reflecting sequential unfolding peaks. Force peaks were well described by the worm-like-chain model (red line with Lc (nm)), using a persistence length of 0.4 nm. The curves shown are representative of a total of more than 200 adhesives curves obtained using 5 independent tips and 5 sample preparations. (C) Superposition of 25 typical force curves showing that the last three or four force peaks in particular are reproducibly observed. (D) Histograms of contour length Lc (n = 165) of the different peaks with Gaussian fit and statistics (mean ± SD). (E,F) Bivariate color-coded contour plots of Lc-ΔLc pairs for every individual unfolding event. Blue and red represent low and high frequencies of events, respectively.

Fha30 was modified with an N-terminal 6-His tag and two Cys residues in tandem at the C terminus, and it was co-produced with its transporter FhaC in *Escherichia coli* and purified from culture supernatants. It was grafted to a gold surface through C-terminal Cys residues and picked up and stretched through the N-terminal 6-His tag using an AFM tip derivatized with NTA-Ni^2+^ (Fig. 2A). Recording force-distance (F-D) curves revealed that a small fraction of all recorded curves exhibited an adhesion event (∼2% from a total of more than 10000 curves). A substantial fraction of the adhesives curves (>40%) exhibited a saw-tooth pattern with successive force peaks, suggesting that the polypeptide chain experienced a multi-step unfolding process as a result of force application (Fig. 2B). In those curves, the unfolding process is characterized by an initial series of small force peaks situated in the first 50 nm. These initial forces peaks differ considerably from one curve to another (Fig. 2B), indicating that the first steps of unfolding may be influenced by non-specific interactions of the protein with the surface. The first region of the F-D curve is followed by several peaks of increasing forces above 100 nm. The gradual, non-linear force increases of the extension steps could be fitted with the worm-like chain (WLC) model using only one free parameter: the contour length (Lc) of the stretched portion of the molecule (Fig. 2B). These fits were used to determine the increases in protein contour length (ΔLc), which reflect the lengths of the protein segments that unfold in each consecutive event. The last peak reflects the extension of the polypeptide to a completely stretched state, where rupture of the bound between the His tag and the Ni^2+^-NTA group of the AFM tip occurs (Fig. 2C). The overall contour length (Lc) of 127 nm of Fha30 determined from this last peak is compatible with full extension of the protein assuming that each amino acid contributes 0.4 nm to the contour length of the fully extended polypeptide chain (the recombinant protein is 325 residues long, thus the expected length for the fully extended polypeptide is 0.4×325 = 130 nm). Superposition of independent force-extension curves (see Materials and Methods for details) showed that the three major unfolding peaks were almost always present, supporting a sequential process along which progressive forces hierarchically unfolded increasingly stable regions of the protein (Fig. 2C,D). This behavior is different from that generally observed for the individual domains of multi-domain proteins, such as titin, that unfold in a more concerted manner, in one or two force peaks [7]. Unfolding peaks of Fha30 occurred at applied forces of ≈125 to 250 pN, consistent with the unfolding of domains with βstructures [7] and much higher than for a-helical proteins [8]. Lc values for all the events were summarized in histograms, with Gaussian fits (mean ± SD, n = 165; Fig. 2D) revealing narrow distributions for the three major peaks of unfolding centered around 43±7 nm, 71±3 nm and 97±2 nm, and for the rupture peak at 127±3 nm. Since some force extension curves showed sawtooth patterns with some missing peaks, bivariate diagrams were used to link Lc values with the ΔLc values corresponding to the distances between each peak and the previous one (Fig. 2E,F). Maxima in the frequency of occurrence of the Lc/ΔLc pairs appear in red/yellow. Bivariate plots revealed that the Fha30 unfolding pathway presents two major unfolding peaks at Lc/?Lc values of 71 nm/32 nm and 97 nm/35 nm as well as a rupture peak at 127 nm/30 nm. The rupture peak was analyzed to determine the ?Lc of the last unfolding peak. Our analysis thus reveals that sequential unfolding, one segment after the other, is the most frequent unfolding pathway for Fha30.

### Simulated unfolding

**Figure 3:**
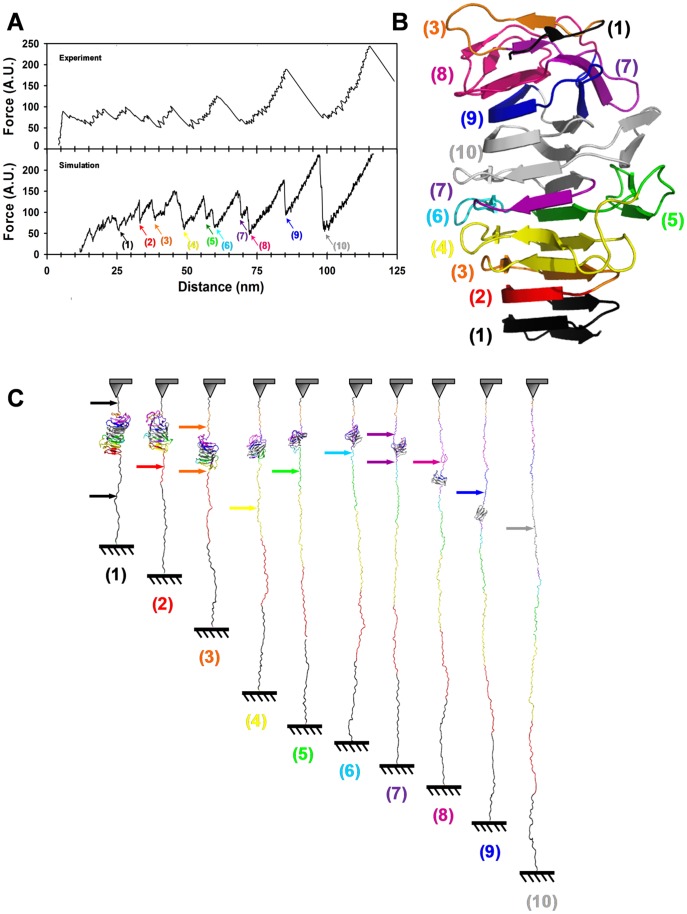
Unfolding of Fha30 using simulated molecular dynamics. (A) Comparison of a typical experimental force curve with a simulated F-D unfolding curve. Steered molecular dynamics simulation was performed using a coarse-grained model of the protein structure without N- and C-terminal tags (304 residues). The colored arrows indicate the positions along the simulation where contact maps were calculated (Fig. S2 in Supporting information). (B) Representation of the Fha30 structure showing the structural elements (colored according to the arrows in panel A) that unfolded in the successive force peaks. (C) Series of snapshots along the SMD unfolding trajectory. The structural elements are colored according to the events observed in the F-D curve and numbered in panel in A. The colored arrows indicate the regions of the protein that unfolded in each of the successive force peaks.

To provide an interpretation for those experiments, we used SMD simulations and we reproduced the extension process by keeping the C terminus fixed and moving the N terminus [26]. In order to perform these calculations in a reasonable time with such a large molecule, we used a structure-based coarse-grained model of Fha30. Therefore, it is difficult to compare the extension speed in the simulations with the experimental extension speed. The simulated F-D profiles obtained at different pulling speeds were similar, indicating a good convergence of the simulations (Fig. S1 in Supporting information). In spite of this limitation, the results from the two techniques were in reasonably good agreement. The simulated force-distance curves reproduced the main pattern of the experimental AFM profiles, exhibiting several small force peaks in the early part of the extension process, followed by several major force peaks of increasing forces (Fig. 3A). SMD simulations, thus, supported a multi-event unfolding reaction, in which regions of different stabilities unfolded successively (Fig. 3A). The different numbers of peaks between experiments and simulation may indicate that experimental unfolding proceeds by larger segments that likely encompass several consecutive *in silico* unfolding steps.

To analyze the unfolding trajectories, a native contact map of Fha30 was built, and its evolution was monitored over time (Fig. S2 in Supporting information). In the early stages of the extension process, the small force peaks represent the sequential unfolding of the R1C and R1B coils (1, in black and 2, in red in Figs. 3B,C) and of the LC1 region, which disappeared in two steps, simultaneously with R1C (in black) and with two strands of R1A (3, in orange). This was followed in the next three peaks by the unfolding of the last strands of R1A together with the C-terminal strands of coil_F (4, in yellow), then unfolding of the last strand of coil_F and of extra-helical element III (5, in green), and finally that of the last strand of coil_E (6, in light blue). In the next step, the N-terminal part of coil_A and the C-terminal part of coil_E, from opposite sides of the molecule, unraveled simultaneously (7, in violet). This was followed by the unfolding in two successive peaks of the second part of coil_A and of extra-helical element I (8, in magenta), and then of coil_B (9, in dark blue). The portion of Fha30 to unfold last (10, in gray) comprised coil_C, coil_D and extra-helical element II. Thus, according to the simulations, mechanical unfolding of Fha30 starts from both ends and spreads gradually towards the centre of the molecule. The simulations thus indicated the presence of a mechanically stable substructure, in agreement with the experimental AFM data that reproducibly showed high-force peaks in the last part of the unfolding process. The simulations define the most stable core as straddling the last part of C1 and the first part of LC2 (in grey in Fig. 3).

### Unfolding of longer variant

**Figure 4:**
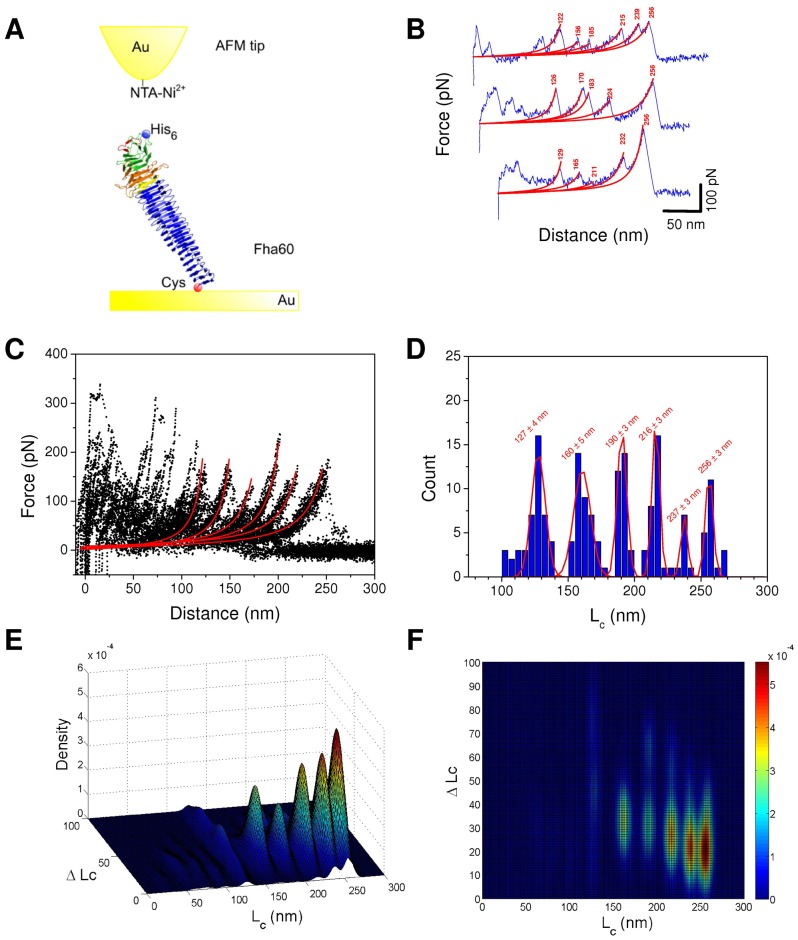
Unfolding of Fha60 by single-molecule AFM. (A) Experimental set-up. (B) Representative force-distance curves obtained by stretching single polypeptides, showing periodic features reflecting sequential unfolding peaks. Force peaks were well described by the WLC model (red line with Lc (nm)), using a persistence length of 0.4 nm. The curves shown are representative of a total of more than 200 adhesives curves obtained using 5 independent tips and 5 sample preparations. (C) Superposition of 30 typical force curves showing that the last six force peaks in particular are reproducibly observed. (D) Histograms of contour length Lc (n = 171) of the different peaks with Gaussian fit and statistics (mean ± SD). (E,F) Bivariate color-coded contour plots of Lc-ΔLc pairs for every individual unfolding event. Blue and red represent low and high frequencies of events, respectively.

To further investigate the unfolding of the first portion of FHA, we analyzed the unfolding pathway of a longer form called Fha60, with 15 additional R1 coils (Fig. 1A). Because Fha60 carrying an N-terminal 6-His tag was poorly secreted by *E. coli* and displayed several degradation bands, the construct was expressed in *B. pertussis* and purified from culture supernatants. Like Fha30, Fha60 thus harbors an N-terminal 6-His tag for interaction with the AFM tip and C-terminal Cys residues for covalent attachment on the gold surface. The recombinant protein was thus stretched through the N-terminal 6-His tag using an AFM tip derivatized with Ni^2+^-NTA (Fig. 4A). Among adhesive curves (which represent ∼1%), only force curves exhibiting rupture lengths compatible with the full extension of the proteins were selected and analyzed (Fig. 4B). Unfolding peaks were fitted with the WLC model (Fig. 4B) and superimposed by using a least squares method (Fig. 4C). Lc values for all the events were summarized in histograms, with Gaussian fits (mean ± SD, n = 171; Fig 4D) revealing narrow distributions for the last six peaks.

The overall rupture length of Fha60 with an Lc value of 256 nm is compatible with full extension of the protein, assuming that each amino acid contributes 0.4 nm to the contour length of the fully extended polypeptide chain. Similarly to Fha30, bivariate diagrams were used to link the Lc values with the ΔLc values corresponding to the distances between two consecutive force peaks (Fig. 4 E,F). Maxima in the frequency of occurrence of Lc/ΔLc pairs appear in red/yellow. Bivariate plots revealed that Fha60 unfolding presents four major peaks at Lc/ΔLc values of 160 nm/36 nm, 190 nm/35 nm, 216 nm/30 nm, and 237 nm/22 nm, and a rupture peak at an Lc/ΔLc value of 256 nm/23 nm. This observation reveals that similar to Fha30, Fha60 unfolds sequentially, one segment after the other.

**Figure 5:**
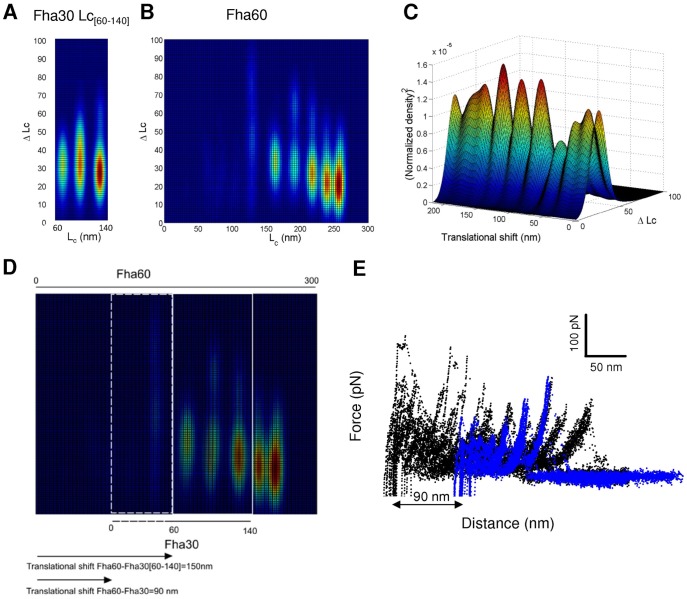
Superposition of the unfolding patterns of Fha30 and Fha60. Comparison between the three major peaks of Fha30 [Lc = 60–140 nm] (A) and the full Fha60 (B) bivariate plots. (C) Normalized product between the two bivariate matrices reveals a maximum for a translational shift of 150 nm. (D) The best alignment between the two proteins occurs for a shift of 90 nm (150 nm minus the first 60 nm from the Fha30 bivariate plot). (E) Superposition of the force extension curves of the two proteins with the calculated best alignment.

Superposition of F-D curves of the two proteins was performed using a correlation analysis between the bivariate plots (Fig. 5A,B). To this end, we extracted the three major peaks of Fha30 (Fig. 5A), and the Fha30 plot was superimposed on the plot of Fha60 and translated progressively to determine the position for optimal superposition (Fig. 5B). Thus, for each relative position of the two plots (called hereafter translational shift), we normalized the corresponding matrix and multiplied the normalized frequency values of Fha30 with those corresponding to Fha60. We obtained a new matrix and we summarized the results as a column by summing up the results for each line. The same procedure was repeated after each translation step of the Fha30 plot relative to the Fha60 plot. The data are displayed as a 3D plot (Fig. 5C). The maxima of this plot reveal that the best alignment is for a translational shift of 150 nm. Since the part of the Fha30 plot used in the superposition procedure starts at an Lc value of 60 nm, the best alignment between the unfolding pathways of Fha30 and Fha60 occurs for a translational shift of 90 nm (Fig. 5D). Thus, using this method, we determined that the two major unfolding peaks of Fha30 correspond to peaks of Fha60 with Lc values of 160 nm and 190 nm (Fig. 5E). Those peaks correspond to substructures endowed with a high mechanical resistance in Fha30, and our analysis indicates that they unfold late in the unfolding pathway of Fha60 as well. The central part of the TPS domain thus appears to be a mechanically resistant portion of FHA. A second region of high mechanical resistance that unfolds in the last steps of the extension is found in Fha60. We also observed that initial force peaks of Fha60 at lower extensions are significantly higher than those of Fha30. We hypothesize that these differences originate from a stronger adsorption of the long molecule on the support, as FHA is known to adhere to abiotic surfaces [27].

## Discussion

The β-helical fold is widespread among secreted microbial proteins [5], [6]. Because proteins in the extracellular milieu are exposed to more variable and harsher conditions than in the intracellular environment, they must adopt structures that are less susceptible to degradation and chemical or mechanical denaturation. Mechanical resistance is particularly important for extracellular proteins involved in interactions with other organisms or with organic or inorganic surfaces. Single-molecule AFM experiments have indicated that proteins rich in βstructure are more resistant to mechanical unfolding than all-α proteins [7], [8]. This may explain why βstructure is common among long extracellular proteins.

In this work, we showed that the βhelix of FHA unfolds at high forces and in a hierarchical manner. In single-molecule force spectroscopy studies performed thus far protein domains unfold in a concerted manner, giving rise to one or two force peaks [7], [8]. By contrast, unfolding of Fha30 and Fha60 induced by pulling apart the N- and C-terminal ends of the proteins exhibits multiple force peaks, revealing substructures of different mechanical resistances that hierarchically unravel under these conditions. Although it has a different fold, the β-structure-rich surface protein OspA of *Borrelia burgdorferi* was reported to also unfold hierarchically [28]. Hierarchical unfolding is a way to ensure that upon forced elongation, weaker substructures unfold first, while more resistant substructures retain their native conformation below a specific force threshold. Partial unfolding might happen when FHA is engaged in attaching the bacterial cell to an epithelial cell surface and is submitted to mechanical forces. The repetitive structure of the βhelix might enable FHA to reacquire its native conformation after release of the mechanical constraint, by using intact portions of the βhelix as templates to reform neighboring coils. Thus, elasticity provided by the solenoid fold is likely to be an important property for adhesive functions. It will be interesting to analyze the mechanical behavior of FHA subjected to a stress using a cellular system.

SMD and single-molecule AFM approaches concur to indicate that the central part of the FHA TPS domain, which includes most of C1 and the N-terminal part of LC2, unfolds under high mechanical forces and in the last part of the unfolding pathway of Fha30. The TPS domain is the hallmark feature of TpsA proteins and systematically found at the N terminus [12]–[14]. Although there are two subtypes of TPS domains with only limited sequence identities between them, their structures are highly similar [12]–[14], featuring a parallel, right-handed βhelix flanked by extra-helical elements. The TPS domain is held together by H bonds between neighboring strands in each of the three βsheets and by the stacking of hydrophobic cores of successive coils, as in regular βhelices, but also by networks of molecular bonds between theβhelix and the extra-helical elements and between the extra-helical elements themselves [12]–[14]. Those features might contribute in particular to the mechanical resistance of the TPS central region. Because its structure is well conserved, the latter is likely to form a region of mechanical resistance in all TpsA proteins.

The best fit for superposing the unfolding patterns of Fha30 and Fha60 shows that the two unfolding steps common to both proteins – encompassing most of the TPS domain- occur after a large portion of Fha60 is already unfolded. This is in agreement with the good mechanical resistance of the TPS domain. The unfolding pattern of Fha60 also shows a late force peak before the rupture peak, indicating another region of high mechanical resistance, within the R1 repeats. Steered MD simulations of Fha30 have indicated that the extremities unfold first, and similar observations were made for the mechanical unfolding of OspA, which is also a single-domain β-structure-rich protein [28]. For Fha30 and Fha60, this is most likely explained by the truncation of the βhelix, which decreases the stability of the C-terminal coils. Thus, the first 150 nm of the Fha60 unfolding curve most likely encompass unfolding of the extremities and of a relatively weaker region of the βhelix between two mechanically resistant domains.

The presence of alternating regions of higher and lower mechanical stability may be a relevant property of the βhelix *in vivo*. Several distinct adherence determinants, for sulfated proteoglycans, lactosylceramide, and integrins, have been described in FHA (reviewed in [16]). They have crudely been mapped to several regions in the central portion of the βhelix [29]–[31]. Alternation of mechanically resistant and less resistant regions most likely maximizes both elasticity and resistance for the long FHA adhesin subjected to mechanical tension *in vivo* when it mediates binding to target tissues. Having adherence determinants in the middle part of the βhelix rather than close to its extremities may also be a good strategy to optimize adhesive functions. In addition, the βhelix of FHA starts with the mechanically resistant TPS domain and is followed by a non-β-helical C-terminal domain [17], [18], which may limit unfolding from the extremities.

Among the many advantages of repetitive folds is the ability to evolve rapidly by the addition, deletion, duplication or rearrangement of repeated units, with beneficial consequences such as the potentiality to generate new binding capabilities or to escape the immune system [32]. This work has revealed that the repetitive βhelix fold also confers upon proteins advantageous mechanical properties. These properties will likely apply to many other prokaryotic and eukaryotic proteins that adopt β-helical or related folds and might play key roles in their functions and structural integrity.

## Materials and Methods

### Plasmids

The various constructs were first generated in pUC19, after which all the fragments of interest were introduced into an expression plasmid called pBBItac. All the amplicons and intermediate constructs were sequenced for verification before the final cloning step. To construct pBBItac, *lacI* and the *tac* promoter of pMMB91 [33] were amplified by PCR with NcoI and XbaI restriction sites at the 5’ ends of the oligonucleotide primers, and the amplicon was introduced into the same sites of pBBR1-MCS5 [34]. To produce Fha30 with the desired tags in *E. coli*, the EcoRI-BamHI fragment coding for Fha30 from plasmid pEC102 [12] was introduced into pUC19, and the internal HindIII site in the signal peptide coding sequence was removed by site-directed mutagenesis, yielding pEC138bis. For production of Fha30 with an N-terminal 6-His tag and C-terminal Cys residues, pEC138bis was modified as follows. The EcoRI-NotI fragment of pEC138bis was replaced by a modified version obtained by overlapping PCRs to insert a 6-His-coding sequence after the first codon of mature Fha30. In addition, a linker coding for the sequence GSQAGRRGRERGRCCRS was introduced into the BamH site at the 3’ end of *fha30*. The added segment with a propensity to form an α helix was designed to cap the extremity of the truncated β helix and to introduce 2 Cys codons at the end of the recombinant protein. This plasmid was called pEC138H-LX2C. The EcoRI-HindIII fragment of the latter plasmid was introduced into pBBItac, yielding pTac138H-LX2C.

Fha60 (comprising 287 additional residues compared with Fha30) with the same tags as Fha30 was initially produced in *E. coli*. Briefly, the construct was prepared as follows. A first PCR was performed to amplify a 700-bp NotI-SalI fragment that straddles *fha30* and the following sequence up to the first SalI site of the *fhaB* gene (both restriction sites are naturally present in *fhaB*), and another PCR was performed to amplify the 730 bp of *fhaB* that follow the SalI site, with a BamH site added to the reverse primer. A triple ligation was then performed between pEC138H-LX2C restricted with NotI and BamHI, and the NotI-SalI and SalI-BamHI amplicons, resulting in pEC138HisN-NM2-LX2C. The EcoRI-HindIII fragment was introduced into pBBItac, yielding pTacHisN-NM2-LX2C. However, production of Fha60 in *E. coli* yielded several major bands of proteolysis in the supernatant in addition to the full-length protein, and therefore the production of the same protein was performed in *B. pertussis*. For this, overlapping PCRs were performed to replace the EcoR1-NotI fragment of pCB4 [35] by a fragment harboring the native promoter and the 5’ coding sequence of *fhaB* modified to insert a 6-His coding sequence immediately after the first codon of mature FHA, yielding pJe3. Then, the NotI-HindIII fragment of pEC138HisN-NM2-LX2C was used to replace the NotI-HindIII fragment of pJe3, yielding pJe3NM2-2C. Finally the EcoRI-HindIII fragment of the latter plasmid was introduced into the same site of pBBR1-MCS5, yielding pB5Je3NM2-2C. This plasmid was used to transform BPGR4, a *B. pertussis* strain carrying a deletion of the *fhaB* gene [36].

### Protein production and purification

Fha60 was produced in *B. pertussis* culture supernatants from BPGR4 harbouring pB5Je3NM2-2C and purified by affinity chromatography on Heparin Sepharose [37]. Fha30 was produced in the supernatants of *E. coli* UT5600 co-producing FhaC and purified as described [12]. Several independent preparations of each protein were analyzed by AFM.

### Atomic force microscopy

AFM measurements were performed at room temperature (20°C) in phosphate-buffered saline solutions (PBS; pH 7.2), using a Nanoscope V and Nanoscope VIII Multimode AFM (Bruker Corporation, Santa Barbara, CA) and gold-coated cantilevers (Olympus, Atomic Force, Germany). The spring constants of the cantilevers were measured using the thermal noise method (Bruker Corporation), yielding values ranging from 0.017 to 0.031 N/m. All force measurements were recorded with a loading rate of 10,000 pN/s, calculated by multiplying the tip pulling velocity (nm/s) by the spring constant of the cantilever (pN/nm). Gold-coated surfaces were prepared by coating silicon wafers (Siltronix) by electron beam thermal evaporation with a 5-nm thick chromium layer followed by a 30-nm thick gold layer.

FHA fragments were attached on the gold-coated surfaces using the added C-terminal Cys residues. To this end gold surfaces were cleaned for 15 min by UV and ozone treatment, rinsed with ethanol, dried with a gentle nitrogen flow and incubated for 30 min in PBS buffer containing 10 µg/mL FHA fragments, and finally rinsed with buffer. Gold-coated AFM tips were immersed overnight in ethanol containing 0.05 mM of nitrilotriacetate-terminated (5%) and triethylene glycol-terminated (95%) alkanethiols (Prochimia, Poland). After rinsing with ethanol, the tips were immersed in a 40 mM aqueous solution of NiSO_4_ (pH 7.2) for 1 h and rinsed with buffer. Several surfaces and several tips were used for each protein preparation.

The force peaks of the F-D curves were fitted with the worm-like-chain model (WLC) [38], using a persistence length of 0.4 nm: *F*(*x*) = *k*_B_ *T/l*_p_ [0.25(1– *x*/*L*_c_)^−2^+*x*/*L*_c_–0.25], where L_c_ and l_p_ are the contour length and persistence length of the molecule, respectively, k_B_ is the Boltzmann constant, and T is the absolute temperature.

To analyze data, we selected force-extension curves exhibiting an overall length compatible with a complete unfolding of the protein fragment. For Fha30 and Fha60 we selected force-extension curves exhibiting overall lengths between 110 and 140 nm and between 250 and 275 nm, respectively. In a second step, we fitted all unfolding peaks with the WLC model and aligned the curves using the method of least squares among WLC fits. All data points for Lc were summarized in histograms and fitted using Gaussian model. In text and in the figures, maxima of the Gaussian fittings are expressed as the mean ± standard deviation (SD). To better identify subpopulations, pairs of Lc and ΔLc were plotted on three-dimensional maps. The color-coded third dimension of the maps corresponds to an estimate of the joint probability of ΔLc and Lc obtained by a lineal-diffusion-based adaptive kernel density algorithm with automatic bandwith selection [39].

### Steered molecular dynamics simulations using a coarse-grained model

SMD simulations were performed using a structure-based model of Fha30 as described in [26]. The crystal structure of Fha30 (**PDB ID: 1RWR**) was reduced to a coarse-grained Cα model with the CG force field [40] using the SMOG@ctbp webserver [41]. The constant velocity SMD simulations were performed with the GROMACS package [42]. The C terminus of the protein was fixed, while the N terminus was moved at constant velocity. The force constant of the spring was set to 6 pN.nm^−1^ and the velocity was varied from 1 to 20 nm.ns^−1^. The protocol tested with the I27 domain of titin reproduced the simulated unfolding process described in the literature [7].

## Supporting Information

Figure S1 **Unfolding F-D curves obtained for wt Fha30 at different pulling speeds: 1, 10 and 20 nm.ns^−1^.** The curves are very similar with only slight shifts in the positions of some peaks, indicating a good convergence of the simulations.
(TIF)

Figure S2 **Contact map of Fha30. Each point represents a distance < 0.6 nm in the structure.** Each colored region of the map indicates the structure that unfolded in the force peak preceding the corresponding arrow in Fig. 3a.
(TIF)
