# Supplementary figures and images for: Sequential Unfolding of Beta Helical Protein by Single-Molecule Atomic Force Microscopy
Source: PLoS One. 2013 Aug 29;8(8):e73572. doi: 10.1371/journal.pone.0073572 (PMC3756990; doi:10.1371/journal.pone.0073572)

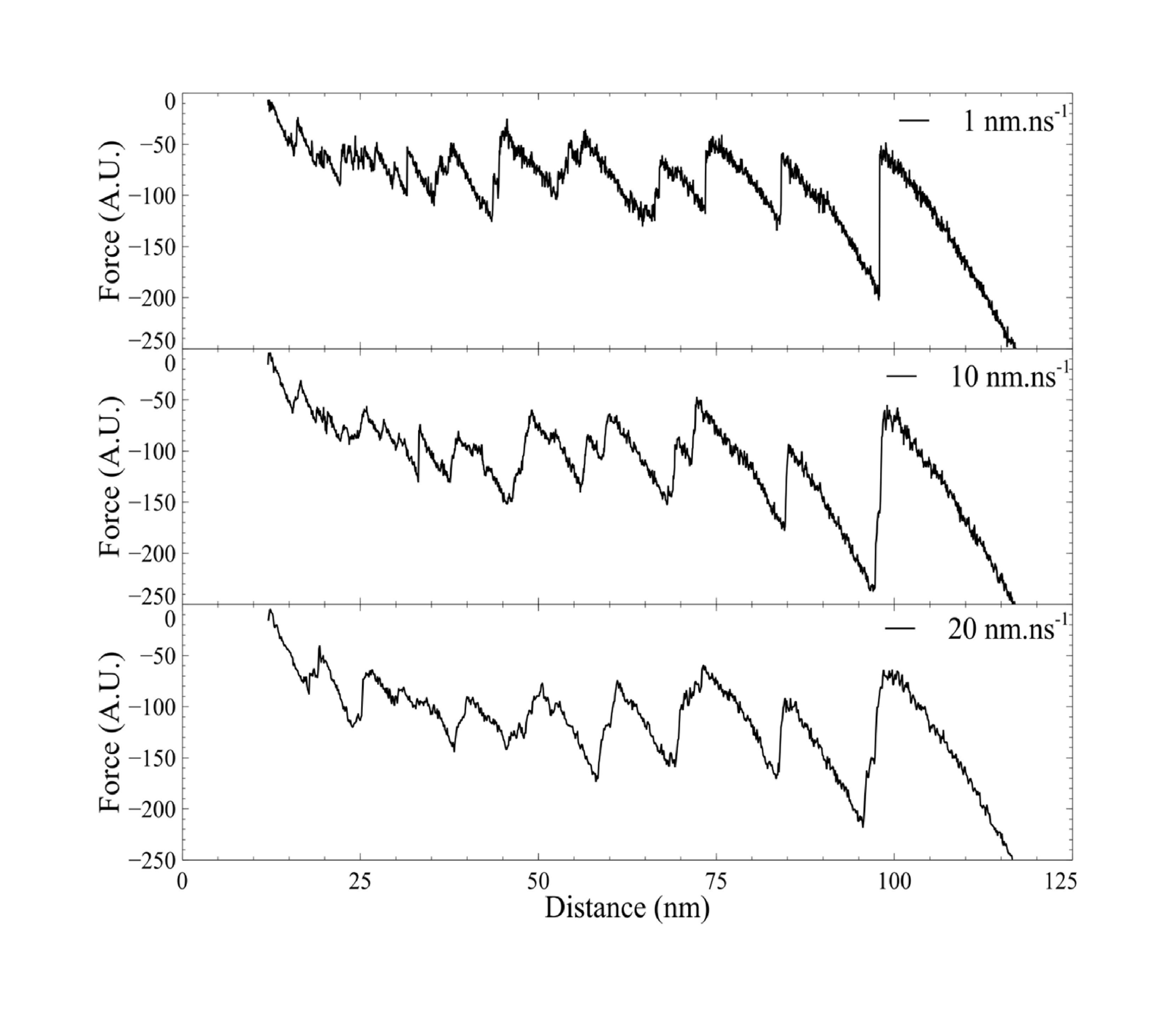

Supplement: Figure S1 — Unfolding F-D curves obtained for wt Fha30 at different pulling speeds: 1, 10 and 20 nm.ns−1. The curves are very similar with only slight shifts in the positions of some peaks, indicating a good convergence of the simulations. (TIF) [file pone.0073572.s001.tif]

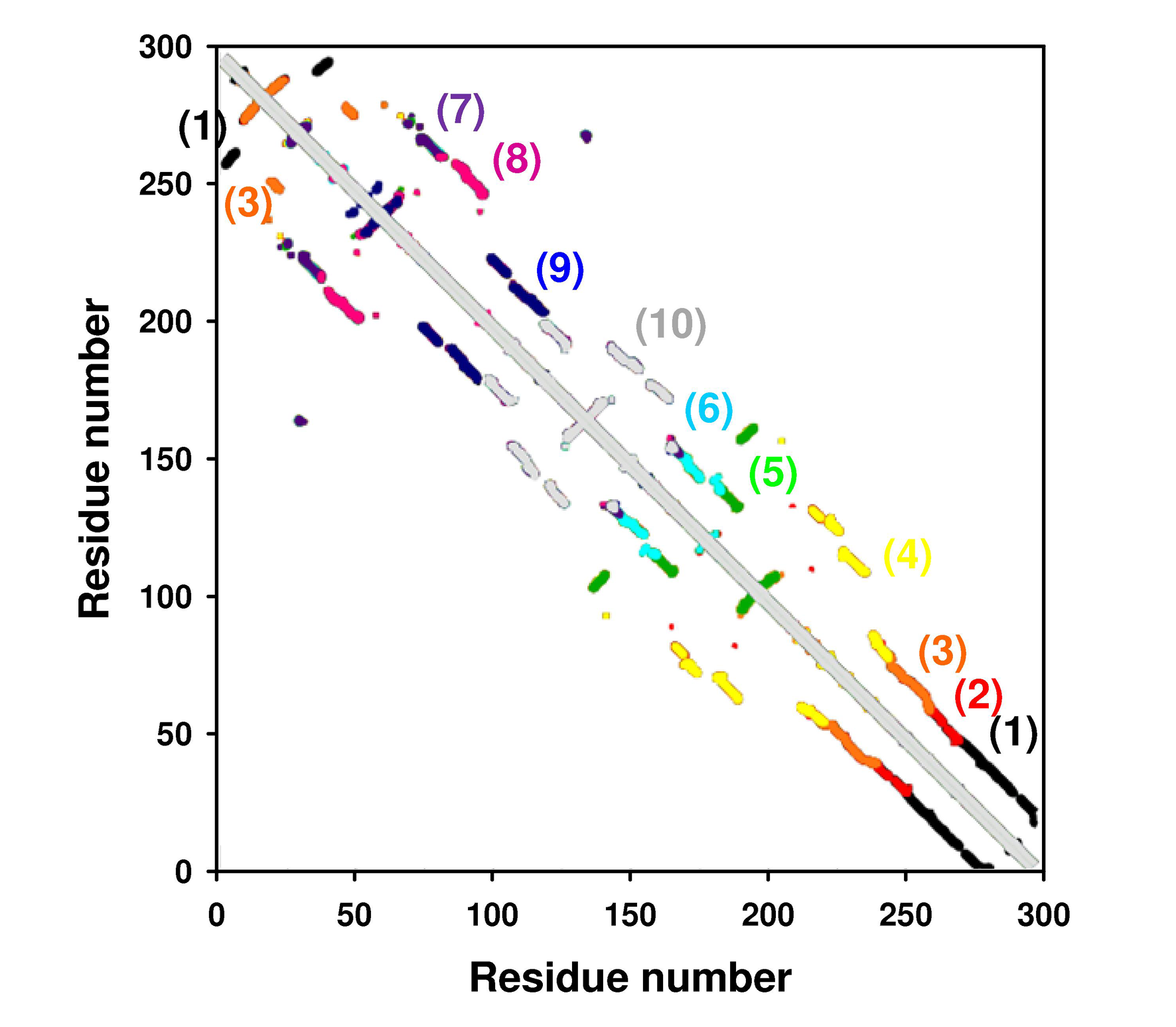

Supplement: Figure S2 — Contact map of Fha30. Each point represents a distance < 0.6 nm in the structure. Each colored region of the map indicates the structure that unfolded in the force peak preceding the corresponding arrow in Fig. 3a. (TIF) [file pone.0073572.s002.tif]
